# Inhibition of DNA Methyltransferases Blocks Mutant Huntingtin-Induced Neurotoxicity

**DOI:** 10.1038/srep31022

**Published:** 2016-08-12

**Authors:** Yanchun Pan, Takuji Daito, Yo Sasaki, Yong Hee Chung, Xiaoyun Xing, Santhi Pondugula, S. Joshua Swamidass, Ting Wang, Albert H. Kim, Hiroko Yano

**Affiliations:** 1 Department of Neurological Surgery, Washington University School of Medicine, St. Louis, MO 63110, USA; 2 Department of Genetics, Washington University School of Medicine, St. Louis, MO 63110, USA; 3 Department of Pediatrics, Washington University School of Medicine, St. Louis, MO 63110, USA; 4 Department of Immunology and Pathology, Washington University School of Medicine, St. Louis, MO 63110, USA; 5 Department of Neurology, Washington University School of Medicine, St. Louis, MO 63110, USA; 6 Department of Developmental Biology, Washington University School of Medicine, St. Louis, MO 63110, USA; 7 Hope Center for Neurological Disorders Washington University School of Medicine, St. Louis, MO 63110, USA

## Abstract

Although epigenetic abnormalities have been described in Huntington’s disease (HD), the causal epigenetic mechanisms driving neurodegeneration in HD cortex and striatum remain undefined. Using an epigenetic pathway-targeted drug screen, we report that inhibitors of DNA methyltransferases (DNMTs), decitabine and FdCyd, block mutant huntingtin (Htt)-induced toxicity in primary cortical and striatal neurons. In addition, knockdown of DNMT3A or DNMT1 protected neurons against mutant Htt-induced toxicity, together demonstrating a requirement for DNMTs in mutant Htt-triggered neuronal death and suggesting a neurodegenerative mechanism based on DNA methylation-mediated transcriptional repression. Inhibition of DNMTs in HD model primary cortical or striatal neurons restored the expression of several key genes, including *Bdnf*, an important neurotrophic factor implicated in HD. Accordingly, the *Bdnf* promoter exhibited aberrant cytosine methylation in mutant Htt-expressing cortical neurons. *In vivo*, pharmacological inhibition of DNMTs in HD mouse brains restored the mRNA levels of key striatal genes known to be downregulated in HD. Thus, disturbances in DNA methylation play a critical role in mutant Htt-induced neuronal dysfunction and death, raising the possibility that epigenetic strategies targeting abnormal DNA methylation may have therapeutic utility in HD.

Huntington’s disease (HD) is a progressive and invariably fatal, autosomal-dominant neurodegenerative disease characterized by progressive loss of selective neurons in the striatum and cortex, leading to movement, cognitive, and psychiatric disorders^1^,^2^. Although HD is known to be caused by an abnormal expansion of polyglutamine repeats in the huntingtin (Htt) protein^3^, how the toxic mutant protein drives neuronal dysfunction and death remains poorly understood, and currently, no curative treatment exists for this disease.

Transcriptional dysregulation is an early abnormality in the course of HD progression and has been suggested to represent an underlying pathogenic mechanism for this disease^4^,^5^,^6^. Previous RNA profiling studies using human and mouse HD brains found a number of critical genes are dysregulated, including downregulation of brain-derived neurotrophic factor (*Bdnf*), dopamine D2 (*Drd2*) receptor, and protein phosphatase 1 regulatory inhibitor subunit 1B (*Ppp1r1b* or *Darpp-32*)^7^,^8^,^9^,^10^,^11^,^12^. BDNF is a major neurotrophic factor implicated in HD pathogenesis and plays important roles in a wide range of neuronal functions, from survival and differentiation to synaptic transmission and learning and memory in the adult brain^13^,^14^. The reduction of BDNF-mediated trophic support from cortical neurons to the medium spiny neurons in HD striatum has been suggested to be a major contributor to the striatal neurodegeneration observed in HD^14^,^15^,^16^,^17^.

Epigenetic mechanisms, including covalent chemical modifications of DNA and histones, play an important role in defining gene expression through modulation of chromatin structure and function^18^,^19^,^20^. Recent genome-wide studies using cell and animal models of HD have identified changes in the patterns of several epigenetic modifications, including DNA methylation and histone methylation, ubiquitination, and acetylation, suggesting that epigenetic dysregulation may be responsible for altered gene transcription observed in HD^12^,^21^,^22^,^23^,^24^. However, it is currently not known which epigenetic alterations in HD play causal roles in death of striatal and cortical neurons as well as disease progression.

Methylation of the fifth carbon position of cytosine (5-methylcytosine, 5mC) in CpG dinucleotide is an epigenetic modification that affects multiple biological processes, including embryonic development, X chromosome inactivation, genomic imprinting, and diseases such as cancers^20^,^25^,^26^. DNA methylation in the gene promoter generally leads to genomic silencing. Recently, the role of this epigenetic modification in the nervous system has begun to attract considerable attention. Emerging evidence suggests that DNA methylation-mediated transcriptional regulation is critically involved in normal brain function as well as brain diseases, including learning and memory, synaptic transmission, and psychiatric disorders^27^,^28^,^29^,^30^,^31^,^32^,^33^,^34^. But little is known about the involvement of DNA methylation in the pathogenesis of neurodegenerative diseases, including HD. DNA methylation is catalyzed by members of the DNA methyltransferase (DNMT) family of enzymes, including DNMT1, DNMT3A, and DNMT3B. Whereas DNMT1 is essential for the maintenance of DNA methylation patterns in the newly synthesized strand during DNA replication, DNMT3A and DNMT3B are required for de novo DNA methylation^20^,^30^. Interestingly, DNMTs are also highly expressed in non-dividing neurons; DNMT1 and DNMT3A are the major DNMTs expressed in postmitotic neurons of the brain^35^,^36^,^37^. How these DNMTs modulate transcription of neuronal genes and contribute to the function and survival of postmitotic neurons remains poorly understood in the context of healthy and diseased brains. Given the altered patterns of several epigenetic modifications in mouse and cell models of HD^12^,^21^,^22^,^23^, identification of the key epigenetic pathway(s) that dominantly drive mutant Htt-induced transcriptional alterations and cytotoxicity in neurons remains an important unanswered question.

Here we demonstrate, through an unbiased epigenetic drug library screen using a primary neuron model of HD, that pharmacological inhibitors of DNMTs effectively protect neurons from mutant Htt-induced toxicity. We also demonstrate that knockdown of DNMT1 or DNMT3A inhibits mutant Htt-induced neuronal toxicity in culture. Inhibition of DNMTs in primary neuron models of HD restored the expression of critical genes known to be downregulated in HD, including *Bdnf*. Consistent with this finding, increased levels of DNA methylation were found in the promoter of *Bdnf* in mutant Htt-expressing neurons. Furthermore, pharmacological inhibition of DNMTs in HD transgenic mice upregulated the transcription of key striatal genes *in vivo*. These findings demonstrate that DNA methylation plays a functionally important role in mutant Htt-induced transcriptional dysregulation and neurotoxicity, raising the possibility that manipulations of the DNA methylation pathway might represent an attractive new therapeutic strategy to attenuate HD neurodegeneration.

## Results

### DNA demethylating agents protect neurons from mutant Htt-induced cytotoxicity

**Figure 1:**
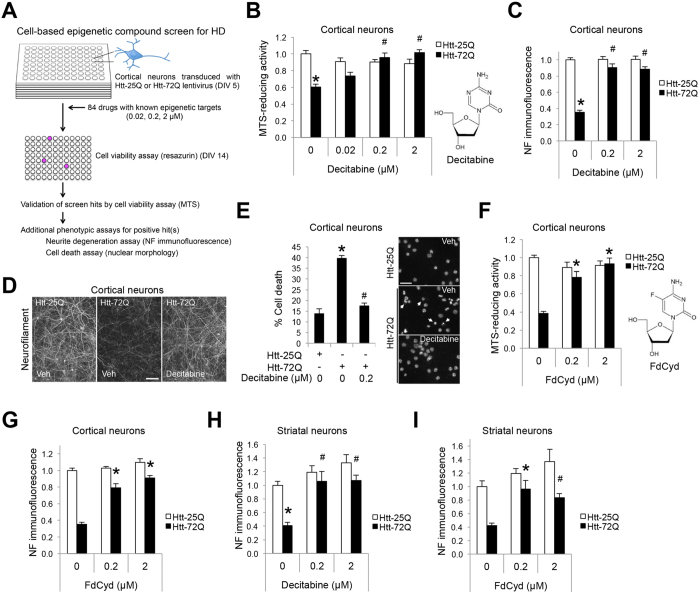
DNMT inhibitors, decitabine and FdCyd, protect neurons from mutant Htt-induced toxicity in culture. (**A**) Schematic of epigenetic drug library screen using a primary neuron model. (**B**) DIV 5 cortical neurons transduced with Htt-expressing lentivirus were treated with decitabine or DMSO (=0 μM), and subjected to MTS assay at DIV 14. Decitabine increased the viability of Htt-72Q-expressing neurons (ANOVA, *P < 0.0001 vs. Htt-25Q (0 μM), ^#^*P* < 0.0001 vs. Htt-72Q (0 μM), n = 18). (**C**) Cortical neurons processed as in (**B**) were fixed at DIV14 and subjected to NF immunofluorescence. Immunofluorescence intensity was quantified. Decitabine blocked Htt-72Q-induced neurite degeneration (ANOVA, *P < 0.0001 vs. Htt-25Q (0 μM), ^#^*P* < 0.0001 vs. Htt-72Q (0 μM), n = 11–24). (**D**) Representative NF immunofluorescence images of transduced neurons treated with decitabine (0.2 μM) or vehicle in (**C**). Bar, 100 μm. (**E**) (Left) Cortical neurons processed as in (**C**) were subjected to nuclear staining (Hoechst 33342). Cell death was assessed by nuclear morphology. Decitabine blocked Htt-72Q-induced cell death (ANOVA, *P < 0.0001 vs. Htt-25Q, ^#^*P* < 0.0001 vs. Htt-72Q (0 μM), n = 16). (Right) Representative nuclear images of transduced neurons. Arrows show examples of condensed or fragmented nuclei, indicating dead cells. Bar, 50 μm. (**F,G**) Cortical neurons transduced as in (**B**) were treated with FdCyd and subjected to MTS assay (**F**) or NF immunofluorescence (**G**) at DIV 14. FdCyd increased the viability of Htt-72Q-expressing neurons (ANOVA, *P < 0.0001 vs. Htt-72Q (0 μM), n = 11–24) (**F**). FdCyd protected neurons from Htt-72Q-induced neurite degeneration (ANOVA, *P < 0.0001 vs. Htt-72Q (0 μM); n = 12–24) (**G**). (**H,I**) DIV 4 striatal neurons were transduced and treated with the indicated DNMT inhibitor. Seven days later, neurons were fixed and subjected to NF immunofluorescence. Decitabine and FdCyd protected neurons from mutant Htt-induced neurite degeneration (ANOVA, *P < 0.0001 vs. Htt-25Q (0 μM), ^#^*P* < 0.0001 vs. Htt-72Q (0 μM), n = 13–17 (**H**); *P = 0.0002 and ^#^*P* = 0.003 vs. Htt-72Q (0 μM), n = 9–16 (**I**)). Data are presented as mean + SEM.

To identify the critical epigenetic pathways that contribute to the death of mutant Htt-expressing neurons, we performed an epigenetic drug screen using a library composed of 84 epigenetic compounds with known targets, including writers, erasers, and readers of the epigenetic code (Fig. 1A and Table S1). As epigenetic gene regulation is a cell-type specific mechanism, we used a physiologically relevant, postmitotic cortical neuron culture system for this screen. In this system, lentivirus-mediated expression of the exon 1-encoded N-terminal fragment of mutant Htt (Htt-72Q with a 72 glutamine repeat), but not wild-type (WT) Htt (Htt-25Q), induces neurotoxicity^38^. The N-terminal short fragments of mutant Htt, which can be generated in cells by proteolytic cleavage of the full-length Htt or alternative splicing, is known to be more cytotoxic than the full-length protein and is expressed in HD patients^39^,^40^,^41^,^42^,^43^,^44^. In the drug screen, the viability of Htt-expressing cortical neurons was determined by the resazurin (Alamar Blue) assay, a quantitative measurement of mitochondrial metabolic activity, which correlates with cell viability. Following validation assays of possible screen “hits” using the MTS assay, we identified the cytosine nucleoside-analog DNA methyltransferase (DNMT) inhibitor decitabine, as the most effective drug in our mutant Htt neuroprotection screen (Fig. 1B and S1A). Remarkably, decitabine exhibited nearly full neuroprotection at 0.2 μM in our HD system (Fig. 1B). Decitabine, also known as 5-aza-2′-deoxycytidine or Dacogen^TM^ (DAC), is a U.S. Food and Drug Administration (FDA)-approved drug and has been used clinically for the treatment of cancers, including myelodysplastic syndrome (MDS) and acute myeloid leukemia (AML)^26^,^45^, but not for neurodegenerative diseases. We further verified the protective effects of decitabine in mutant Htt-expressing primary cortical neurons using two additional assays: neurite degeneration by quantifying the loss of neurofilament (NF) immunofluorescence intensity, an early marker of neuronal toxicity (Fig. 1C,D), and cell death by scoring condensed or fragmented nuclei (Fig. 1E). Since decitabine is an epigenetic agent that affects gene expression, we confirmed that decitabine does not decrease the expression of Htt-72Q in our system compared to vehicle control by qRT-PCR (Fig. S2A). We then tested if treatment with decitabine affects the burden of mutant Htt aggregates—an HD pathological hallmark—in primary cortical neurons using an antibody that preferentially detects mutant Htt aggregates (Fig. S2B–D). By both immunofluorescence and immunoblot analyses, we found that decitabine could decrease the levels of mutant Htt aggregates in Htt-72Q-expressing neurons. Given that misfolded and aggregated Htt may interfere with several important biological functions in neurons, its reduction may contribute to neuroprotection by decitabine.

To verify the effect of DNMT inhibition against mutant Htt toxicity, we next performed similar experiments with three other well-characterized nucleoside-analog DNMT inhibitors, 5-fluoro-2′-deoxycytidine (FdCyd), 5-azacytidine (azacitidine, 5-AC, Vidaza^TM^), and zebularine. The latter two drugs are ribonucleoside analogs, which target primarily RNA rather than DNA, and small fractions of these drugs can be converted to their deoxyribose form in cells, thereby leading to inhibition of DNA methylation^46^,^47^. 5-azacytidine, like decitabine, is a FDA-approved, potent anti-cancer drug that has been used for the treatment of MDS and AML. FdCyd was developed by the National Cancer Institute and is currently being investigated in ongoing clinical trials in solid tumors. Interestingly, the deoxyribonucleoside analog FdCyd, but not the ribonucleoside analogs, zebularine and 5-azacytidine, demonstrated neuroprotective effects against mutant Htt-induced toxicity in primary cortical neurons in cell viability and neurite degeneration assays (Fig. 1F,G, and S3A–C), suggesting that the deoxyribonucleoside form of DNMT inhibitors, which act directly through DNA, exerts neuroprotective activity in HD neurons.

To test if DNMTs play a role in the HD striatal neurons, one of the most severely affected cell types in the disease, we established a cultured striatal neuron model, in which the N-terminal exon-1 fragment of mutant or WT Htt was expressed by lentiviral infection. Strikingly, treatment with decitabine or FdCyd also attenuated mutant Htt-induced striatal neurite degeneration (Fig. 1H,I). Similar to cortical neurons, 5-azacytidine did not inhibit mutant Htt-induced neurite degeneration in striatal neurons (Fig. S3D). Together, results utilizing two disease-relevant neuronal cell types suggest that DNMTs play an important role in mutant Htt-induced neurodegeneration.

### Reduced DNMT3A or DNMT1 expression protects neurons from mutant Htt-induced toxicity

**Figure 2:**
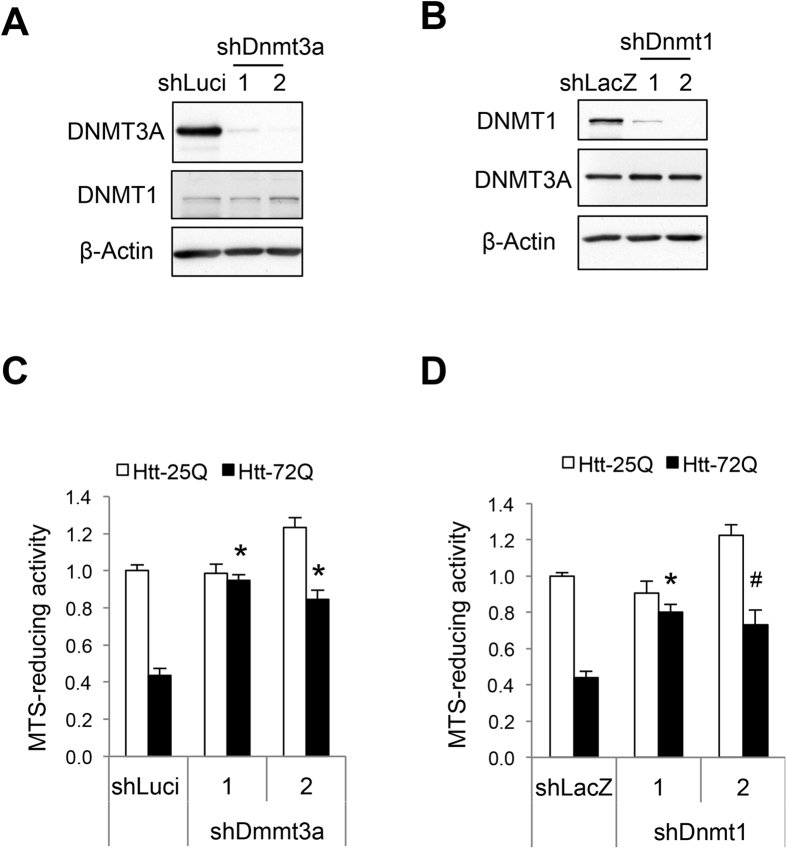
Lentivirus-mediated knockdown of DNMT3A or DNMT1 in primary cortical neurons attenuates mutant Htt-induced toxicity. (**A**) DIV 5 cortical neurons were transduced with two *Dnmt3a* shRNA (1 and 2) or control luciferase (Luci) shRNA lentivirus; 5 days later, cell lysates were subjected to immunoblotting using indicated antibodies. (**B**) Cortical neurons transduced with two *Dnmt1* shRNA (1 and 2) or control LacZ shRNA lentivirus were subjected to immunoblotting as in (**A**). (**C**) DIV 5 cortical neurons were co-transduced with Htt-expressing lentivirus along with *Dnmt3a* or control shRNA lentivirus and were subjected to MTS assay at DIV14. Knockdown of DNMT3A in mutant Htt-expressing neurons was neuroprotective (ANOVA, *P < 0.0001 compared to Htt-72Q plus control RNAi, n = 17–20 wells per group, 5 independent experiments). (**D**) Cortical neurons co-transduced with Htt lentivirus and *Dnmt1* or control shRNA lentivirus were subjected to MTS assay as in (**C**). Knockdown of DNMT1 in mutant Htt-expressing neurons was neuroprotective (ANOVA, *P < 0.0001 and ^#^*P* = 0.0001 compared to Htt-72Q plus control RNAi, n = 11–15 wells per group, 4 independent experiments). Data are presented as mean + SEM in (**C**,**D**).

Because inhibition of DNMTs by decitabine and FdCyd rescued neurons from mutant Htt-induced toxicity, we next determined if genetic inhibition of DNMTs by RNA interference (RNAi) attenuates neuronal death in the mutant Htt context. Among members of the DNMT family, postmitotic neurons in the brain are known to highly express DNMT3A and DNMT1^27^,^35^,^36^. Knockdown of either DNMT3A or DNMT1 protein by lentiviral delivery of two distinct short hairpin RNAs (shRNAs) for each DNMT significantly increased the viability of mutant Htt-expressing cortical neurons (Fig. 2A–D). Knockdown of DNMT3A or DNMT1 did not decrease the levels of the other DNMT’s mRNA or protein (Fig. 2A,B, and S4A,B), demonstrating specificity of the shRNAs used. These findings indicate that both DNMT3A and DNMT1 are required for mutant Htt-induced neuronal death. Taken together, these results demonstrate that DNA methylation plays a causal role in mutant Htt-induced neurotoxicity, likely by repressing the transcription of genes important for neuronal survival and function.

### Inhibition of DNMTs restores *Bdnf* gene expression in mutant Htt-expressing cortical neurons

**Figure 3:**
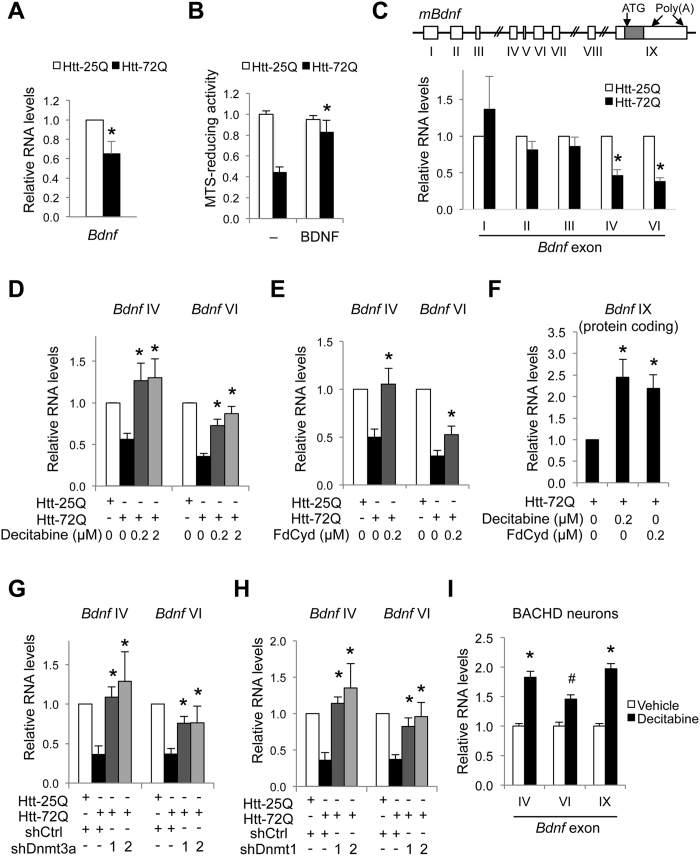
Inhibition of DNMTs restores the expression of *Bdnf* exon IV and VI transcripts in primary cortical neurons. (**A**) DIV 5 cortical neurons were infected with Htt lentivirus. RNA was harvested 5 days later and subjected to qRT-PCR for total *Bdnf* (coding exon IX) using *β-actin* and 18S rRNA as reference genes. Htt-72Q decreased the expression of total *Bdnf* transcripts (Mann-Whitney *U* test, *P = 0.008 vs. Htt-25Q, n = 5). (**B**) Cortical neurons transduced as in (**A**) were cultured with recombinant BDNF (50 ng/ml) and subjected to MTS assay at DIV 14. BDNF increased the viability of Htt-72Q-expressing neurons (ANOVA, *P < 0.0001 vs. Htt-72Q with vehicle, n = 9–15). (**C**) (Top) Schematic of the mouse *Bdnf* locus. White boxes, non-coding exons; gray box, coding exon. (Bottom) qRT-PCR was performed as in (**A**) using exon-specific *Bdnf* primers. Htt-72Q decreased the expression of exon IV and VI transcripts (Mann-Whitney *U* test, *P = 0.008 vs. Htt-25Q, n = 5). (**D**–**F**) Cortical neurons transduced with Htt lentivirus were treated with indicated DNMT inhibitor or vehicle and processed as in (**C**). gRT-PCR was performed using *β-actin* and *Hprt* as reference genes. Both decitabine and FdCyd increased the expression of *Bdnf* exon IV, VI, and IX transcripts in Htt-72Q-expressing neurons (ANOVA, *P < 0.005 vs. Htt-72Q plus vehicle, n = 5–7 (**D**); *P < 0.05 vs. Htt-72Q plus vehicle, n = 5 (**E**); *P < 0.05 vs. Htt-72Q plus vehicle, n = 7 (**F**)). (**G**,**H**) Cortical neurons were co-transduced with lentiviruses expressing Htt and indicated shRNA and processed as in (**D**). Knockdown of DNMT3A or DNMT1 restored the expression of *Bdnf* exon IV and VI (ANOVA, *P < 0.05 vs. Htt-72Q plus vehicle, n = 4 (**G**); *P < 0.01 vs. Htt-72Q plus vehicle, n = 4 (**H**)). (**I**) Primary cortical neurons from BACHD mice were treated with decitabine (0.2 μM) or vehicle for 3.5 days. qRT-PCR was performed using *β-actin* as a reference gene. Decitabine increased expression of *Bdnf* exon IV, exon VI, and IX transcripts (unpaired *t*-test, *P < 0.0001 and ^#^*P* = 0.0012 vs. vehicle treated). Data are presented as mean + SEM.

BDNF is a major neurotrophic factor involved in fundamental brain processes, including neuronal survival, synaptic plasticity, and learning and memory. *Bdnf* mRNA and protein levels were found to be decreased in the brains of human HD patients and mouse models, which is thought to contribute to HD pathology^11^,^12^,^15^. Consistent with these observations, *Bdnf* expression was reduced by mutant Htt expression in primary cortical neurons (Fig. 3A). Addition of recombinant BDNF protein in the culture medium was sufficient to rescue cortical neurons from mutant Htt-induced toxicity (Fig. 3B), suggesting an important role for BDNF in the survival of mutant Htt-expressing neurons. Using *Bdnf* as a model gene, we next focused on determining if *Bdnf* transcriptional repression could be rescued by manipulating DNA methylation in mutant Htt-expressing neurons. As the *Bdnf* gene has a complex structure with multiple noncoding exons and a common protein coding exon, we first examined the differential expression of major exon-specific *Bdnf* transcripts in primary cortical neurons. Each noncoding exon has an independent promoter, and the expression of the exon-specific transcript is differentially regulated in response to diverse extracellular stimuli and signaling events (schematic in Fig. 3C)^48^,^49^,^50^. Mutant Htt-expressing cortical neurons exhibited decreased expression of *Bdnf* mRNA at a time before neurons begin to die, specifically *Bdnf* exon IV- and VI-containing transcripts, compared to control neurons expressing WT Htt-25Q or the empty vector (Fig. 3C, data not shown), consistent with a previous observation^51^. The reduction of these transcripts is clinically relevant since they have also been found to be decreased in HD model mouse brains and human HD postmortem brain^14^,^15^,^52^.

Next, to test the hypothesis that abnormal DNA methylation contributes to the downregulation of *Bdnf* mRNA, we examined if pharmacological inhibition of DNMTs could rescue the expression of *Bdnf* exon IV and VI-containing mRNAs in mutant Htt-expressing cortical neurons by qRT-PCR analysis (Fig. 3D,E). Intriguingly, both decitabine and FdCyd restored the levels of *Bdnf* exon IV and VI transcripts at doses effective for neuroprotection (Fig. 3D,E). These DNMT inhibitors also increased the levels of the common coding exon IX transcript (total *Bdnf* mRNA) in mutant Htt-expressing cortical neurons (Fig. 3F). Consistent with the effects of DNMT inhibitors on *Bdnf* transcription, knockdown of DNMT3A or DNMT1 in mutant Htt-expressing cortical neurons using two shRNAs targeting each DNMT reversed the mutant Htt-triggered decrease in *Bdnf* exon IV and VI mRNAs (Fig. 3G,H). These results suggest that both DNMTs contribute to downregulation of *Bdnf* mRNA in HD neurons.

To verify these findings using an alternative HD model system, we next determined if decitabine could upregulate *Bdnf* mRNA expression in primary cortical neurons derived from bacterial artificial chromosome (BAC)-mediated HD transgenic (BACHD) mice, which express full-length mutant Htt^53^. BACHD mice exhibit progressive motor deficits and late-onset selective neuropathology in the cortex and striatum^53^. Inhibition of DNMTs by decitabine in BACHD mouse cortical neurons increased *Bdnf* exon IV- and VI-containing as well as total *Bdnf* (exon IX) mRNAs by qRT-PCR (Fig. 3I), supporting the findings obtained using neurons expressing the N-terminal fragment of mutant Htt (Fig. 3D). Collectively, these results suggest that DNMT inhibition exhibits neuroprotective effects in the context of mutant Htt in part through the upregulation of *Bdnf*.

### Mutant Htt increased the levels of DNA methylation in the *Bdnf* exon IV regulatory region in primary cortical neurons

**Figure 4:**
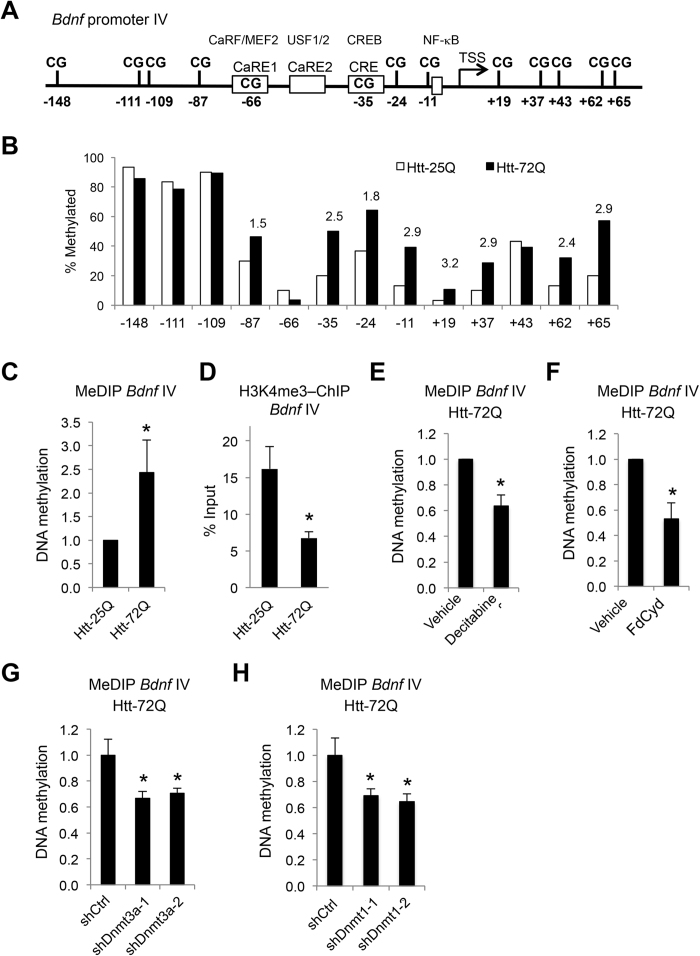
Mutant Htt increases the levels of DNA methylation at *Bdnf* exon IV regulatory region in primary cortical neurons. (**A**) Schematic of the mouse *Bdnf* exon IV regulatory region near the TSS. The positions of CpG sites are indicated relative to the TSS. (**B**) DIV 5 primary cortical neurons were infected with lentivirus expressing Htt-25Q or Htt-72Q exon 1 fragment; 5 days later, genomic DNA was purified and subjected to bisulfite sequencing analysis. The data show percentage of cytosine residues that were methylated in Htt25Q- and Htt-72Q-expressing neurons. Increased DNA methylation was found in mutant Htt-expressing neurons compared to WT Htt-expressing neurons. 28–30 clones from 7 independent experiments were analyzed (See Figure S5A for the bisulfite sequencing data from each clone). The number above the black bar (Htt-72Q) represents the fold changes in methylated cytosine relative to the white bar (Htt-25Q) at the indicated position. (**C**) Genomic DNA was purified from primary cortical neurons transduced as in (**B**) and subjected to MeDIP with anti-5-mC antibody followed by qPCR. The levels of 5-mC in the exon IV promoter region was higher in Htt-72Q-expressing neurons compared to that in Htt-25Q neurons (Mann-Whitney *U* test, *P < 0.05, n = 6). (**D**) Cortical neurons were transduced as in (**B**) and 5 days later were subjected to ChIP with anti-H3K4me3 antibody. H3K4me3 levels in the exon IV promoter region were lower in Htt-72Q-expressing neurons compared to Htt-25Q neurons. (unpaired *t*-test, *P < 0.05, n = 5). (**E,F**) Cortical neurons were processed and subjected to MeDIP as in (**C**) using Htt-72Q-expressing neurons treated with DNMT inhibitors (0.2 μM) or DMSO. Treatment with decitabine or FdCyd decreased levels of 5-mC in *Bdnf* promoter IV region (Mann-Whitney *U* test, *P = 0.002, n = 6 (**E**); *P = 0.008, n = 5 (**F**)). (**G,H**) Cortical neurons co-transduced with lentiviruses expressing Htt-72Q and indicated shRNA were processed as in (**C**) for MeDIP. Knockdown of DNMT3A or DNMT1 could decrease the levels of *Bdnf* promoter IV methylation (ANOVA, *P < 0.05 vs. Htt-72Q plus vehicle, n = 6). Data are presented as mean + SEM in (**C–H**).

Because *Bdnf* exon IV and VI transcripts in mutant Htt-expressing cortical neurons are increased by DNMT inhibition (Fig. 3D,E,G–I), we next test the hypothesis that mutant Htt stimulates DNA methylation in the promoter regions of these two exons, leading to repression of these transcripts. The *Bdnf* promoter IV harbors several transcription factor binding sites, including calcium responsive elements, CaRE1, CaRE2, and CRE (schematic in Fig. 4A) and is activated in response to various extracellular stimuli *in vivo*^54^,^55^,^56^. The levels of DNA methylation in the *Bdnf* exon IV regulatory region that contains 13 CpG sites (base pairs −148 to +65 relative to the transcriptional start site (TSS)) was assessed by bisulfite conversion followed by DNA sequencing, a widely used technique to measure levels of cytosine methylation on specific genomic regions with single CpG resolution. Among the 13 CpGs sites examined, methylation of eight CpGs between base pairs −87 to +65 was increased (1.5- to 3.2-fold) by mutant Htt expression (Fig. 4B and S5A). Three CpG sites located between base pairs −148 and −109, including the previously reported methyl CpG binding protein 2 (MeCP2) binding site at the position −148^55^,^56^, exhibited robust methylation with no significant difference between WT and mutant Htt-expressing neurons (Fig. 4B). Thus the bisulfite sequencing results revealed that mutant Htt-expressing neurons exhibit an overall increase in the levels of cytosine methylation, compared to WT Htt-expressing neurons, in the regulatory region of the *Bdnf* exon IV surrounding the TSS (Fig. 4B and S5A). The increased levels of cytosine methylation (5-mC) in the region was independently confirmed by methylated DNA immunoprecipitaiton (MeDIP), which uses a specific antibody against 5-mC (Fig. 4C). These data suggest that mutant Htt expression downregulates *Bdnf* exon IV transcript via increased DNA methylation of the promoter. In contrast, similar bisulfite sequencing analysis for the regulatory region of *Bdnf* exon VI containing 17 CpG sites displayed little if any DNA methylation in both WT Htt-25Q and mutant Htt-72Q-expressing cortical neurons (Fig. S5B), indicating that *Bdnf* promoter VI is not a direct target of DNA methylation but is indirectly suppressed by events initiated by aberrant DNA methylation in mutant Htt-expressing neurons.

DNA methylation-mediated gene repression is generally associated with a closed chromatin structure, which is induced by cooperation with altered histone modifications^57^. By chromatin immunoprecipitation (ChIP) analysis we found that the mutant Htt-triggered increase in DNA methylation is associated with decreased trimethylation at lysine 4 of histone H3 (H3K4me3), a transcriptionally active histone mark, in the promoter region of *Bdnf* exon IV in mutant Htt-expressing primary cortical neurons compared to WT Htt-expressing neurons (Fig. 4D). Together, these results illustrate that mutant Htt-induced increases in DNA methylation are associated with loss of active open chromatin in this region, consistent with mutant Htt-induced transcriptional repression of *Bdnf* exon IV.

Next, to further support the hypothesis that decreased *Bdnf* exon IV transcription by mutant Htt is the consequence of changes in DNA methylation at this locus, we examined whether inhibition of DNMTs in mutant Htt-expressing primary cortical neurons decreases DNA methylation in the regulatory region of *Bdnf* exon IV by MeDIP-qPCR. We found that inhibition of DNMTs by decitabine or FdCyd reversed the increase in DNA methylation triggered by mutant Htt (Fig. 4C,E,F). Consistently, knockdown of either DNMT3A or DNMT1 decreased the levels of DNA methylation in this region (Fig. 4G,H). Together, our results suggest that altered DNA methylation drives the repression of *Bdnf* transcription in HD neurons and demonstrate that two distinct DNMTs in neurons are both required for the mutant Htt-induced increase in DNA methylation in the *Bdnf* regulatory region.

### Decitabine reactivates expression of key striatal genes in a primary striatal neuron model of HD

**Figure 5:**
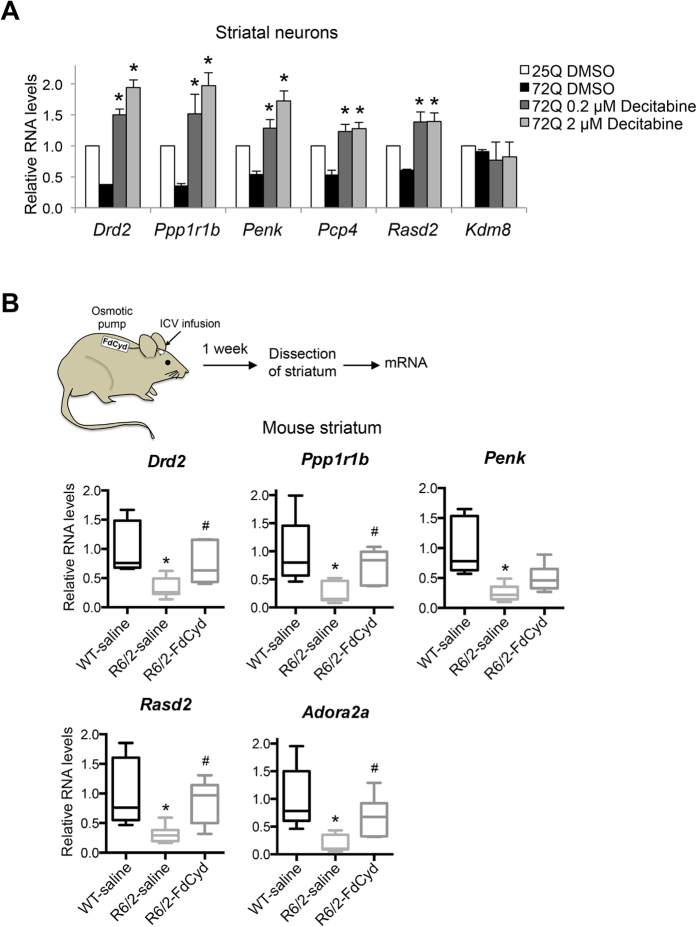
DNMT inhibitors reactivate striatal gene expression in mutant Htt-expressing primary neurons and R6/2 HD mouse brain *in vivo*. (**A**) DIV 5 mouse primary striatal neurons were infected with lentivirus expressing Htt-25Q or Htt-72Q exon1 fragment; 5 days later, RNA was prepared and subjected to qRT-PCR analysis. *β-actin* and *Hprt* were used as reference genes. Decitabine restored the expression of downregulated genes in mutant Htt-expressing striatal neurons (ANOVA, *P < 0.05, n = 3 compared to Htt-72Q plus vehicle. Similar results were observed when Htt-72Q-expressing neurons were treated with FdCyd (data not shown). Data are presented as mean + SEM. (**B**) (Top) Procedure for the treatment of mice with FdCyd. A mini-osmotic pump containing FdCyd (0.1 mM in saline) was implanted subcutaneously on the back of mice at 6 weeks of age, and the drug was infused into the right ventricle through a stereotactically placed catheter. One week later, the striatum was dissected for qRT-PCR analysis. ICV, intracerebroventricular. (Bottom) FdCyd was delivered into R6/2 or WT mouse brain by icv infusion at 6 weeks of age. Saline was used as control. One week after drug infusion was initiated, RNA was extracted from the striatum and subjected to qRT-PCR analysis. *β-actin* was used as a reference gene. Levels of *Drd2*, *Ppp1r1b, Rasd2, and Adora2a* mRNA were restored in R6/2 mice after FdCyd treatment. FdCyd treatment showed a trend towards increasing *Penk* RNA in R6/2 striatum. (ANOVA, *P < 0.005 compared to WT–saline, ^*#*^*P* < 0.05 compared to R6/2–saline, n = 7–9 mice per group). The vertical bars represent the range of values.

Given the neuroprotective effect of decitabine in mutant Htt-expressing striatal neurons (Fig. 1H), we next tested if DNMT inhibition with decitabine restores the expression of other genes that are known to be downregulated in HD. Consistent with previous gene expression studies using human and mouse HD striatum^7^,^8^,^12^,^58^,^59^, qRT-PCR analyses demonstrated that mutant Htt expression in primary striatal neurons triggers robust changes in gene expression, including downregulation of dopamine receptor D2 (*Drd2),* protein phosphatase 1, regulatory (inhibitor) subunit 1B *(Ppp1r1b*, also known as *Darpp-32*), preproenkephalin (*Penk), Purkinje cell protein 4 (Pcp4)*, and RASD family, member 2 *(Rasd2,* also known as *Rhes*) (Fig. 5A). Thus our culture system faithfully reproduces key gene expression changes observed in HD *in vivo*. These transcriptional changes were detected before mutant Htt neurons exhibit significant neurite degeneration (data not shown), suggesting that mutant Htt-induced transcriptional changes contribute to striatal neurodegeneraiton. Inhibition of DNMTs by decitabine dramatically increased the expression of these downregulated transcripts (Fig. 5A). This reactivation of gene expression was specific because the mRNA levels of lysine (K)-specific demethylase 8 (*Kdm8*) were unchanged by decitabine treatment. Together these results demonstrate that the inhibition of DNA methylation can restore gene expression, which is deficient in HD neurons, suggesting that abnormal DNA methylation plays a critical role in transcriptional dysregulation in HD striatal and cortical neurons.

### Pharmacological inhibition of DNMTs in HD mouse brains upregulates the expression of key striatal genes *in vivo*

**Figure 6:**
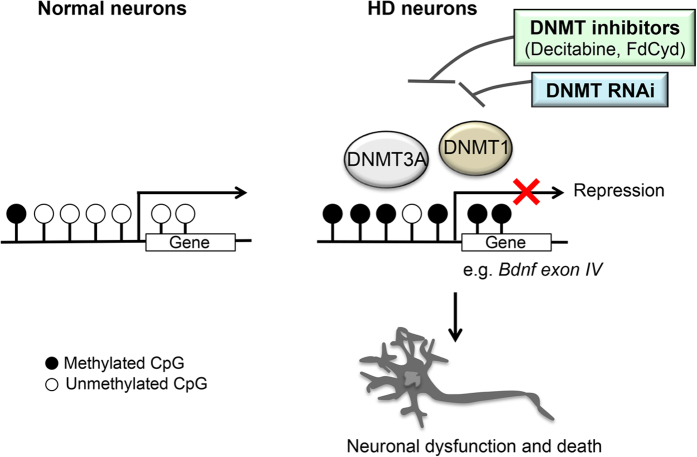
A model for the role of DNA methylation in HD neurodegeneration. Inhibition of DNMTs in HD neurons by pharmacological inhibitors (decitabine or FdCyd) or RNAi blocks mutant Htt-induced neurotoxicity as well as transcriptional repression of key genes, such as *Bdnf, Drd2, Ppp1r1b, and Adora2a*. The DNA methylation pathway may thus play an important role in HD neurodegeneration.

We next determined if DNMT inhibition could restore the expression of genes downregulated in HD *in vivo* using R6/2 HD mouse, a well-characterized transgenic mouse model expressing an N-terminal mutant Htt fragment^60^. This mouse model exhibits robust phenotypes with early disease onset and short life span and recapitulates the altered expression of a number of genes observed in HD patients, including *Drd2* and *Ppp1r1b* in the striatum early in the course of disease progression^7^,^12^,^60^,^61^. Although decitabine has been reported to cross the blood-brain barrier^62^,^63^, the cytosine nucleoside analog DNMT inhibitors, including decitabine and FdCyd, are known to be degraded rapidly by cytidine deaminase in the liver (*in vivo* half life of decitabine <20 min)^62^, indicating that systemic administration may not be an effective strategy for drug delivery to the brain. We therefore chose intracerebroventricular (icv) administration using an Alzet osmotic pump, which provides continuous infusion of drug at a consistent rate from a subcutaneous pump (Fig. 5B). Although FdCyd when tested structurally similar to decitabine, we found that, whereas decitabine lost its *in vitro* neuroprotective activity after 11 days of pre-incubation at 37 °C in saline, FdCyd fully maintained its neuroprotective activity even after 45 days of pre-incubation (Figure S6A,B), suggesting that FdCyd is chemically more stable than decitabine at 37 °C *in vitro* and is better suited for drug delivery with osmotic pumps. The instability of decitabine *in vitro* has been reported previously^64^,^65^. We therefore used FdCyd to determine the effect of DNMT inhibition on gene expression in R6/2 brain. One week after the implantation of Alzet osmotic pumps filled with FdCyd or saline in R6/2 mice or WT littermates, RNA was prepared from the striatum (Fig. 5B). Striatal expression of several key mRNAs, *Drd2*, *Ppp1r1b, Rasd2, Adora2a, and Penk* mRNAs, was found to be downregulated in HD mouse striatum compared to control animals (Fig. 5B), consistent with previous reports in human and mouse HD striata as well as in our HD model striatal neurons (Fig. 5A)^7^,^8^,^12^. Infusion of FdCyd in R6/2 brains significantly upregulated the expression of striatal *Drd2*, *Ppp1r1b, Rasd2, and Adora2a* mRNAs and also showed a trend towards increasing *Penk* mRNA (Fig. 5B), indicating that pharmacological inhibition of DNMTs can correct transcriptional deficiencies in HD mouse brain. Together, these results suggest that DNA methylation plays an important role in transcriptional alterations in HD and potentially, neuronal dysfunction and death *in vivo* (See a model in Fig. 6).

## Discussion

In this study, we have demonstrated that pharmacological or genetic inhibition of DNMTs substantially attenuates mutant Htt-induced transcriptional dysregulation and neurotoxicity in primary cortical and striatal neurons. We have also provided evidence that aberrant promoter methylation contributes to a reduction in *Bdnf* expression in mutant Htt-expressing cortical neurons. Given the neuroprotective effects of exogenous BDNF in HD model cortical neurons, blockade of DNMTs may protect neurons from mutant Htt-induced death in part through upregulation of *Bdnf* gene expression. Remarkably, *in vivo* experiments demonstrated that treatment of HD mice with DNMT inhibitor FdCyd could reverse the transcriptional repression of key striatal genes in HD mouse brain. Together, we provide evidence that DNA methylation in HD is a critical epigenetic mechanism, which underlies mutant Htt-induced transcriptional alterations and neurodegeneration, raising the possibility that the DNA methylation pathway might represent a new therapeutic target for HD.

Previous studies have shown that a large number of genes are dysregulated in the brains of HD patients and various mouse models^5^,^6^,^7^,^8^,^9^,^10^,^11^,^12^. Recent genome-wide analysis as well as candidate gene approaches using cell and mouse models of HD have found altered patterns of several epigenetic modifications^66^,^67^, including acetylation, ubiqutination, and methylation (H3K4me3 and H3K9me3) of histones^12^,^21^,^23^,^24^,^68^,^69^,^70^ as well as methylation (5-mC) and hydroxymethylation (5-hmC) of DNA^22^,^71^. Therefore, abnormal chromatin state may be a critical driver for neurodegeneration in HD. Although the causal role of these epigenetic modifications in vulnerable neurons in HD remains unknown, our unbiased drug library screen with 84 chemical compounds, which target known epigenetic pathways, suggests that DNA methylation-mediated gene silencing plays a dominant role in triggering neuronal death.

In primary cortical neuron models, we found that mutant Htt induces increased DNA methylation in the regulatory region of *Bdnf* exon IV, which is associated with transcriptional repression and a reduction in the transcriptionally active H3K4me3 mark. Additionally, inhibition of DNMTs by pharmacological inhibitors or RNAi could rescue the expression of *Bdnf* exon IV mRNA in mutant Htt-expressing primary cortical neurons (Fig. 3D,E,G–I), suggesting that the mutant Htt-triggered increase in DNA methylation in this region directly causes transcriptional repression. Multiple previous studies have suggested that DNA methylation and histone modifications exhibit crosstalk and cooperate in the regulation of gene expression^57^,^72^. Understanding the epigenetic hierarchy downstream of mutant Htt in neurons in relation to RNA expression represents an important future direction.

How mutant Htt promotes DNA methylation at specific gene loci at the molecular level remains a significant open question. Possible mechanisms include: 1) mutant Htt expression in neurons increases the levels of DNMT expression, 2) mutant Htt enhances the activity of DNMTs, 3) mutant Htt facilitates the recruitment of the DNA methylation machinery to specific genomic regions, and/or 4) mutant Htt increases 5-mC levels by decreasing DNA demethylation activity in neurons. The first mechanism, however, is unlikely since we have found that mutant Htt does not significantly increase the mRNA or protein levels of DNMT1 or DNMT3A in primary cortical neurons (Y.P. and H.Y., unpublished data). The second and third mechanisms are reasonable possibilities and may be caused by aberrant protein-protein interactions and/or abnormal posttranslational modifications of DNMTs downstream of mutant Htt. Regarding the fourth possible mechanism, the recent discovery of the ten-eleven translocation (TET) family of enzymes that promote DNA demethylation by converting 5-mC to 5-hmC and the abundance of TET proteins and 5-hmC in brain has highlighted DNA methylation as a dynamically regulated process important for brain function^30^,^73^,^74^,^75^,^76^,^77^. Therefore, whether mutant Htt increases 5-mC levels on repressed genes by inhibiting the DNA demethylation pathway in HD is an interesting question.

BDNF deficiency in the brains of HD patients and mouse models has been suggested to play a crucial role in the development of the disease^14^,^15^,^16^,^17^,^78^,^79^. However the mechanisms underlying downregulation of exon-specific *Bdnf* transcripts, in particular, exon IV and VI in HD neurons remains largely unknown. We focused on *Bdnf* as a model gene to test the hypothesis that mutant Htt represses neuronal gene expression through promoter hypermethylation. Our results show that mutant Htt expression increases cytosine methylation in the regulatory region of *Bdnf* exon IV and that inhibition of DNMTs decreases the methylation and reactivates exon IV transcription, supporting the idea that increased DNA methylation plays a causal role in repression of *Bdnf* transcription in HD. In contrast, the regulatory region of *Bdnf* exon VI, appears not to be directly regulated by DNA methylation, suggesting instead that indirect mechanisms are initiated by aberrant DNA methylation in the control of the *Bdnf* exon VI repression. Several approaches to increase BDNF-mediated trophic support in HD brain are being performed in ongoing clinical trials and preclinical studies^80^,^81^,^82^,^83^. Our results suggest that manipulation of DNA methylation may offer a new therapeutic approach to increase neuronal BDNF expression in HD brain.

The reduction of either DNMT1 or DNMT3A by RNAi is sufficient to block transcriptional changes and neuronal death induced by mutant Htt (Fig. 2A–D and 3G,H), suggesting that both DNMTs are required to exert mutant Htt-mediated toxicity. Although in dividing cells, the roles of DNMT1 and DNMT3A as maintenance and de novo DNMTs, respectively, are known, the specific roles of the two DNMTs in postmitotic neurons remain undefined, necessitating exploration in future studies. An intriguing previous study demonstrated that double conditional knockout mice lacking DNMT3A and DNMT1 in postnatal forebrain excitatory neurons, but not single ablation of either DNMT3A or DNMT1, exhibited a deficit in synaptic function and learning and memory, suggesting redundant or overlapping roles^32^. Targeting either DNMT1 or DNMT3A in adult neurons therefore may attenuate mutant Htt-induced neurotoxicity with minimal side effects in regard to normal synaptic functions in the brain. It has been demonstrated that the nucleoside analog DNMT inhibitors, such as decitabine and FdCyd, must first be incorporated into DNA to exert their DNMT inhibitory activity^46^,^65^,^84^. In dividing cells, drug incorporation occurs during DNA synthesis. The mechanism of action of these DNMT inhibitors in non-dividing postmitotic neurons, however, still remains unclear, although it is possible that the base excision repair pathway contributes to the incorporation of nucleoside-analog DNMT inhibitors. Improved molecular understanding of the action of decitabine and FdCyd may identify potential “hot spots” of incorporation in the neuronal genome, providing relevant information regarding specific gene targets undergoing active methylation in the HD epigenome. Integrating genome-wide DNA methylation and transcriptional changes associated with DNMT inhibition in future studies will identify the key gene targets of DNMT inhibition-induced demethylation in HD neurons.

Finally, the findings from the current study immediately suggest that inhibition of DNMTs might ameliorate HD phenotypes *in vivo*, which will be the subject of important future experiments. Improved understanding of the epigenetic gene regulation in HD neurons will provide important foundational knowledge for the development of therapeutic strategies targeting DNA methylation abnormalities in HD.

## Methods

### Antibodies and reagents

Mouse monoclonal anti-neurofilament (NF) (165 kDa) (clone 2H3, Developmental Studies Hybridoma Bank) was used for immunofluorescence. Mouse monoclonal anti-β-actin (sc-47778, Santa Cruz Biotechnology), rabbit monoclonal anti-DNMT1 (D63A6, Cell Signaling Technology, Inc.), and rabbit polyclonal anti-DNMT3A (sc-20703, Santa Cruz Biotechnology) antibodies were used for immunoblotting. Mouse monoclonal anti-Htt (EM48) antibody^85^ (MAB5374, Millipore) was used for immunofluorescence and immunoblotting. Decitabine was purchased from Cayman Chemical (11166) and LC laboratories (D-3899). 5′fluoro-2′deoxycytidine (FdCyd) was purchased from Sigma (F5307) and Santa Cruz Biotechnology (sc-252267). These drugs were confirmed to exhibit similar effects regardless of the source.

### Plasmids

Lentiviral expression plasmids containing Htt exon1-25Q (Htt-25Q) and Htt exon1-72Q (Htt-72Q) constructs under the control of the mouse *PGK* (*Pgk1*) promoter (mPGK-Httex1-25Q and mPGK-Httex1-72Q) were kindly provided by D. Krainc (Northwestern University, Chicago, IL). Lentivirus-based Dnmt3a RNAi and Dnmt1 RNAi constructs (pLKO.1-puro), developed at the Broad Institute of MIT and Harvard, were obtained (Sigma-Aldrich). The oligo sequences in the shRNA vectors targeted Dnmt3a and Dnmt1 are as follows:

pLKO.1-Dnmt3a#1 (TRCN0000039034): CCGGCCAGATGTTCTTTGCCAATAACTCGAGTTAT TGGCAAAGAACATCTGGTTTTTG; pLKO.1-Dnmt3a#2 (TRCN0000039035): CCGGGCAGACCAACATCGAATCCATCTCGAGATGGATTCGATGTTGGTCTGCTTTTTG; pLKO.1-Dnmt1#1 (TRCN0000219081): GTACCGGATCTATGGAAGGTGGTATTAACTCGAGTTAATACCACCTTCCATAGATTTTTTTG; pLKO.1-Dnmt1#2 (TRCN0000225698): CCGGTATATGAAGACCTGATCAATACTCGAGTATTGATCAGGTCTTCATATATTTTTG.

### Primary neuron cultures, lentiviral transduction drug treatments

Mouse primary cortical and striatal neurons from embryonic day (E) 15.5 Swiss Webster mouse fetuses (Taconic) were first plated in the minimal essential medium (MEM) containing 10% FBS, 0.37% glucose, 1 mM sodium pyruvate, 2 mM glutamine, 20 U/ml penicillin and 20 μg/ml streptomycin, for 3 h and then maintained in serum-free Neurobasal medium (Life Technologies) containing NeuroCult^TM^ SM1 neuronal supplement (STEMCELL Technologies), 0.5 mM glutamine and 25 μM glutamate for the first 3 d in a humidified incubator (37 °C in 5% CO_2_). Half of the medium was replaced with Neurobasal medium with SM1 and 0.5 mM glutamine every 3 days. Primary cortical neurons plated on 96-well flat clear bottom black plates (Corning #3904) at 4 × 10^4^ cells/well were infected with Htt exon1 expression lentivirus (Htt-25Q or Htt-72Q) or control empty vector lentivirus at 5 days *in vitro* (DIV 5). Primary striatal neurons plated on 96-well plates at 1 × 10^5^ cells/well were infected with Htt exon1 expression lentivirus at DIV 4. Viral copy number was adjusted for transduction of neurons on the basis of titer measured using the Lenti-X qRT-PCR titration kit (Clontech), and equal numbers of viral particles of Htt-25Q and Htt-72Q expressing lentiviruses were used for transduction. For the experiments to test effects of DNMT inhibitors, neurons were treated with inhibitors six hours after Htt lentiviral infection. One half of the media was changed every 3 days with media containing new drug. In knockdown experiments in Htt-expressing neurons, primary cortical neurons were cotransduced with Htt-expressing lentivirus and *Dnmt* shRNA or control shRNA lentivirus at DIV 5. pLKO.1-TRC1-luciferase (Luci) and pLKO.1-TRC2-LacZ were used as control for RNAi with pLKO.1-TRC1-Dnmt3a and pLKO.1-TRC2-Dnmt1, respectively. Lentiviral particles were prepared by transfecting 293LE cells with the lentiviral plasmid of interest along with packaging plasmid psPAX2 and envelope plasmid pCMV-VSVG as described previously^86^. Four days after transfection, viruses in the conditioned media were collected and purified using Lenti-X Concentrator (Clontech). Primary cortical neurons from BACHD mouse embryos (E15.5) were individually plated into separate wells and treated at DIV 4 with decitabine or vehicle for 3.5 days.

### Measurements of cell viability/cytotoxicity in primary neurons

Primary cortical neurons grown in a 96-well plate were transduced with Htt-expressing lentiviruses at DIV 5 and assessed for mitochondrial metabolic activity at 9 days post-infection (DIV 14) using 3-(4,5-dimethylthiazol-2-yl)-5-(3-carboxymethoxyphenyl)-2-(4-sulfophenyl)-2H-tetrazolium (MTS) (Promega) per manufacturer’s instructions. MTS-reducing activity was normalized for each condition to Htt-25Q lentiviruses treated with vehicle or cotransduced with control RNAi lentivirus (=1). Experiments were performed in 3 or more wells per experiment in three to five independent experiments.

For the measurement of neurofilament (NF) immunofluorescence intensity, cortical and striatal neurons cultured in a 96-well plate were fixed in 4% paraformaldehyde (PFA) in PBS for 20 min nine and seven days after Htt lentiviral infection, respectively, permeabilized with 0.1% Triton X-100 in PBS for 15 min at room temperature, and subjected to indirect immunofluorescence with anti-NF (2H3) primary antibody and Alexa Fluor 568-conjugated goat anti-mouse IgG secondary antibody (Life Technologies). Images of Alexa Fluor 568-labeled were captured (nine random fields per well) using an Operetta high-content imaging system (PerkinElmer) with a 20 × objective lens. Following image background subtraction, the NF immunofluorescence intensity was quantified using an ImageJ-based macro. Image capture and quantification of Htt (EM48) immunofluorescence intensity were performed as described for those of NF. In this quantification analysis, we confirmed that the number of cells in a cultured well are similar among mutant Htt-expressing neurons with or without DNMT inhibitor treatment, by counting the number of nuclei (Hoechst 33342) in the images used for quantification: Htt-72Q neurons treated with vehicle (299 ± 7.8 cells), decitabine (299 ± 4.6 cells), n = 18 wells from 6 independent experiments, and therefore the data reflect EM48 intensity per cell. For the quantification of cell death, primary cortical neurons grown in a 96-well plate were infected with Htt lentivirus at DIV 5 and fixed nine days after infection as described above. Cell nuclei were labeled with Hoechst 33342 (Life Technologies), and neurons were assessed in a blinded fashion for cell death by scoring condensed or fragmented nuclei. Experiments were performed in 4 to 6 wells per experiment in three independent experiments. About 300 nuclei from three random fields in a well were counted.

### Drug library screen

Epigenetic drug screen was performed using a primary cortical neuron model of HD with a drug library composed of 84 compounds (Table S1), among which 80 drugs are purchased from Cayman Chemical (Epigenetic Screening Library Item No 11076) and four drugs, SGC0946, EPZ5676, EPZ6438, and GSK126, were obtained from Xcessbio Biosciences Inc. Mouse primary cortical neurons were plated on 96-well flat clear bottom black plates (Corning #3904). WT or mutant Htt exon1 fragment (Htt-25Q or Htt-72Q)-expressing lentiviruses are infected at DIV 5 as described above. The 84 compounds were added to the media at DIV 6 one day after Htt lentiviral infection with three different doses (0.02, 0.2, 2 μM) for each compound at triplicates. DMSO was used as control. One half of the media containing compounds or DMSO were changed every three days to maintain the compounds’ activity. At DIV 14, the viability of neurons was determined by resazurin (Alamar blue) assay, a quantitative measurement of mitochondrial metabolic activity. The screen was fully automated and performed in the High-Throughput Screening Center in Washington University School of Medicine. Any possible plate effects were determined using control plates treated with DMSO and used for normalization. Screen hits were validated by MTS assay.

### HD transgenic mice and drug administration

R6/2 mice, which carry the promoter sequence and exon 1 of a mutant human *HTT* gene, were obtained from JAX (Stock No: 002810) (Bar Harbor, ME), and a colony was maintained by breeding R6/2 males with B6CBAF1 females (JAX). PCR genotyping was performed using a primer set (CGGCTGAGGCAGCAGCGGCTGT and GCAGCAGCAGCAGCAACAGCCGCCACCGCC) as described previously^60^. To maintain mice carrying the same number of CAG repeats, a second PCR analysis was also conducted using a primer set amplifying across the CAG repeats (ATGAAGGCCTTCGAGTCCCTCAAGTCCTTC and GGCGGCTGAGGAAGCTGAGGA). BACHD mice on the C57BL/6J background, which were generated by the laboratory of X. William Yang (University of California, Los Angeles)^53^,^87^, were obtained from the CHDI Foundation. All live vertebrate experiments were performed in compliance with the US National Institutes of Health Guide for the Care and Use of Laboratory Animals. Animal protocols were approved by the Institutional Animal Care and Use Committees of Washington University. To determine the effect of DNMT inhibitor on gene expression in HD mouse brain *in vivo*, FdCyd (0.1 mM in saline) was directly administered into 6 week-old R6/2 mice and control littermates by stereotactic intracerebroventricular (icv) infusion using Alzet mini-osmotic pump (DURET Corporation, MODEL 2001; 1.0 μl/h, 7 days) and the brain infusion kit 3 (DURET Corporation, #0008851). Saline was used as control. One week later, the striatum was dissected and processed for qRT-PCR analysis. The CAG repeat length of R6/2 mice used for the *in vivo* gene expression analysis was determined by Laragen Inc. (Culver City, CA) using tail DNA and was approximately 210.

### Quantitative reverse transcription PCR (qRT-PCR)

RNAs were isolated from cultured neurons 5 days after infection of Htt lentiviruses and mouse brain using the RNeasy Plus Mini Kit (QIAGEN) and RNeasy Plus Universal Mini Kit (QIAGEN), respectively. Reverse transcription was performed with High-Capacity cDNA Reverse Transcription Kit (Applied Biosystems). qPCR was performed using Power SYBR Green PCR Master Mix (Applied Biosystems) on CFX Connect Real-Time System (Bio-Rad). *β-actin* and hypoxanthine phosphoribosyltransferase I (*Hprt) or 18S rRNA* were used as reference genes for data normalization unless otherwise stated. Relative mRNA levels were calculated using the **∆∆**Cq method. Sequences of the primers used for qRT-PCR analysis are listed in Table S2A.

### Bisulfite conversion and sequencing

Genomic DNA was extracted from cells using QIAamp DNA Mini Kit (QIAGEN) and subjected to bisulfite conversion using EZ DNA Methylation-Lightning™ Kit (Zymo Research) according to the manufacturer’s instructions. Gene regulatory regions for *Bdnf* exons IV and VI were PCR amplified using ZymoTaq™ DNA Polymerase (Zymo Research) from the bisulfite-converted DNA templates. The PCR fragments were subcloned into the pCR2.1-TOPO vector using TOPO TA cloning kit (Life Technologies) and sequenced with M13 primer (Genewiz). The primers used for PCR amplification of the bisulfite-converted genomic DNA are listed in Table S2B.

### Methylated DNA immunoprecipitation (MeDIP)

Genomic DNA was isolated from primary cortical neurons using QIAamp DNA Mini Kit (QIAGEN) and fragmented by sonication using Bioruptor (Diagenode). 5-mC-containing DNA fragments were enriched from one μg of the sonicated genomic DNA by immunoprecipitation (IP) with mouse monoclonal anti-5mC antibody (Eurogentec, # BI-MECY-0100) as described previously^88^. IP and 10% input DNA samples were purified using MinElute PCR Purification Kit (QIAGEN) and subjected to qPCR with *Bdnf* promoter IV and *Gapdh* primers to measure the enrichment of the DNA fragment containing the *Bdnf* promoter IV region. Primer sequences are provided in Table S2C. The percentage input was calculated by first normalizing IP to input DNA using the formula (2^[(Ct(10% input) – 3.32) − Ct(IP)]^ × 100) as described previously^89^. *Gapdh* was used as an internal normalization control.

### Chromatin immunoprecipitation (ChIP)

ChIP assays from mouse primary neurons were performed using Magna ChIP kit (Millipore) and anti-H3K4me3 antibody (Millipore, 17–614). The percentage input was calculated as 2^[(Ct(10% input) – 3.32) − Ct(IP)]^ × 100 and compared between WT and mutant Htt-expressing neurons. Sequences of the primers used to amplify the BDNF promoter IV fragment are listed in Table S2D.

### Statistical analysis

Statistical differences were tested using XLSTAT and GraphPad Prism 6.0. Two-tailed unpaired Student *t* test for two group comparisons or one-way ANOVA with post-hoc tests, the Fisher’s least significant difference (LSD) for comparison among three groups or the Bonferroni analysis for comparison among three or more than three groups. The Mann-Whitney *U* test was used for nonparametric test for comparing two groups. The data presented are from at least three independent experiments.

## Additional Information

**How to cite this article**: Pan, Y. *et al*. Inhibition of DNA Methyltransferases Blocks Mutant Huntingtin-Induced Neurotoxicity. *Sci. Rep.* **6**, 31022; doi: 10.1038/srep31022 (2016).

## Supplementary Material

Supplementary Information
