# Erratum: Inhibition of DNA Methyltransferases Blocks Mutant Huntingtin-Induced Neurotoxicity

**DOI:** 10.1038/srep33766

**Published:** 2016-09-21

**Authors:** Yanchun Pan, Takuji Daito, Yo Sasaki, Yong Hee Chung, Xiaoyun Xing, Santhi Pondugula, S. Joshua Swamidass, Ting Wang, Albert H. Kim, Hiroko Yano

Scientific Reports 6: Article number: 3102210.1038/srep31022; published online: 08 12 2016; updated: 09 21 2016

This Article contains typographical errors. In the Results section under subheading ‘DNA demethylating agents protect neurons from mutant Htt-induced cytotoxicity.’

“Following validation assays of possible screen “hits” using the MTS assay, we identified the cytosine nucleoside-analog DNA methyltransferase (DNMT) inhibitor decitabine, as the most effective drug in our mutant Htt neuroprotection screen (Fig. 1B and S1A).”

should read: 

“Following validation assays of possible screen “hits” using the MTS assay, we identified the cytosine nucleoside-analog DNA methyltransferase (DNMT) inhibitor decitabine, as the most effective drug in our mutant Htt neuroprotection screen (Fig. 1B and S1A,B).”

In the same section under subheading ‘Pharmacological inhibition of DNMTs in HD mouse brains upregulates the expression of key striatal genes *in vivo*’.

“Although FdCyd when tested structurally similar to decitabine, we found that, whereas decitabine lost its *in vitro* neuroprotective activity after 11 days of pre-incubation at 37 °C in saline, FdCyd fully maintained its neuroprotective activity even after 45 days of pre-incubation (Figure S6A,B), suggesting that FdCyd is chemically more stable than decitabine at 37 °C *in vitro* and is better suited for drug delivery with osmotic pumps.”

should read:

“Although FdCyd is structurally similar to decitabine, we found that, whereas decitabine lost its *in vitro* neuroprotective activity when tested after 11 days of pre-incubation at 37 °C in saline, FdCyd fully maintained its neuroprotective activity even after 45 days of pre-incubation (Figure S6A,B), suggesting that FdCyd is chemically more stable than decitabine at 37 °C *in vitro* and is better suited for drug delivery with osmotic pumps.”

In the Methods section under subheading ‘Measurements of cell viability/cytotoxicity in primary neurons.’

“Images of Alexa Fluor 568-labeled were captured (nine random fields per well) using an Operetta high-content imaging system (PerkinElmer) with a 20 × objective lens. Following image background subtraction, the NF immunofluorescence intensity was quantified using an ImageJ-based macro.”

should read:

“Images of Alexa Fluor 568-labeled NF were captured (nine random fields per well) using an Operetta high-content imaging system (PerkinElmer) with a 20 × objective lens. Following image background subtraction, the NF immunofluorescence intensity was quantified using an ImageJ-based macro.”

