# Supplementary material for: Inhibition of DNA Methyltransferases Blocks Mutant Huntingtin-Induced Neurotoxicity
Source: Sci Rep. 2016 Aug 12;6:31022. doi: 10.1038/srep31022 (PMC4981892; doi:10.1038/srep31022)
Supplement: Supplementary Information [file srep31022-s1.pdf]

Supplementary Information

Inhibition of DNA Methyltransferases Blocks Mutant Huntingtin-Induced Neurotoxicity

Yanchun Pan[1], Takuji Daito[1], Yo Sasaki[2], Yong Hee Chung[1], Xiaoyun Xing[2], Santhi Pondugula[3], S. Joshua Swamidass[4], Ting Wang[2], Albert H. Kim[1,5,6,7], and Hiroko Yano[1,2,5,7,*]

[1]Department of Neurological Surgery
[2]Department of Genetics
[3]Department of Pediatrics
[4]Department of Immunology and Pathology
[5]Department of Neurology
[6]Department of Developmental Biology
[7]Hope Center for Neurological Disorders
Washington University School of Medicine, St. Louis, MO 63110, USA

*Correspondence:
Email: yanoh@wudosis.wustl.edu

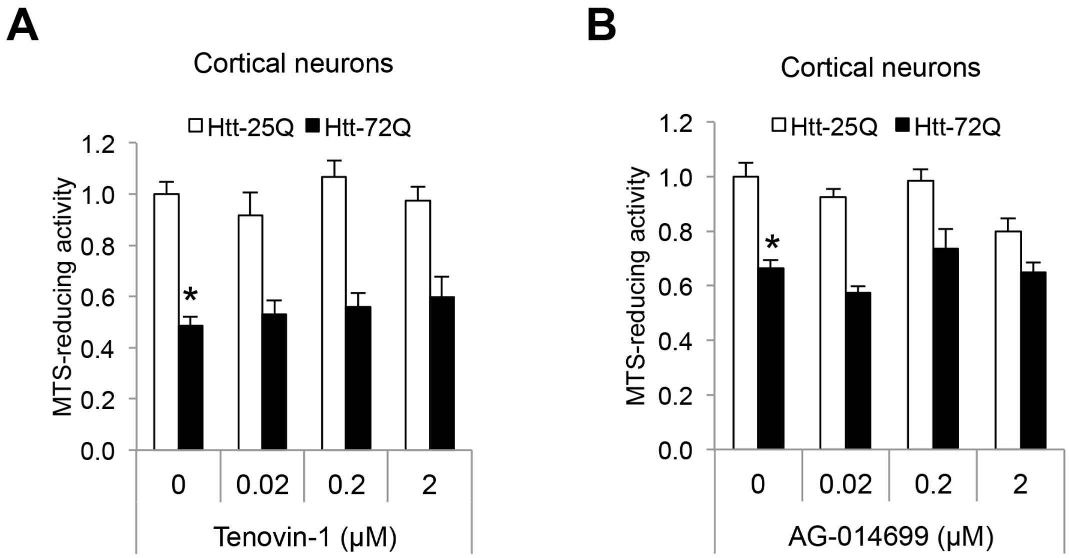

Figure S1. Validation of hits from epigenetic drug screen.

(A) Tenovin-1 (p53 activator), one of the top three positive compounds from the cell-based screen (Figure 1A), was subjected to cell viability assay using MTS. Treatments with tenovin-1 did not significantly increase the viability of mutant Htt-expressing neurons (ANOVA, *$P < 0.0001$ compared to Htt-25Q (0 μM); $P = 0.591$, Htt-72Q (0.02 μM) vs Htt-72Q (0 μM); $P = 0.355$, Htt-72Q (0.2 μM) vs Htt-72Q (0 μM); $P = 0.168$, Htt-72Q (2 μM) vs Htt-72Q (0 μM); n = 6-12 wells per group).

(B) AG-014699 (PARP1 inhibitor), another screen hit, was subjected to cell viability assay as in (A). There is no significant difference in survival between the AG-014699- and vehicle-treated Htt-72Q neurons (ANOVA, *$P < 0.0001$ compared to Htt-25Q (0 μM); $P = 0.142$, Htt-72Q (0.02 μM) vs Htt-72Q (0 μM); $P = 0.254$, Htt-72Q (0.2 μM) vs Htt-72Q (0 μM); $P = 0.781$, Htt-72Q (2 μM) vs Htt-72Q (0 μM); n = 12 wells per groups).

Data are presented as mean + SEM.

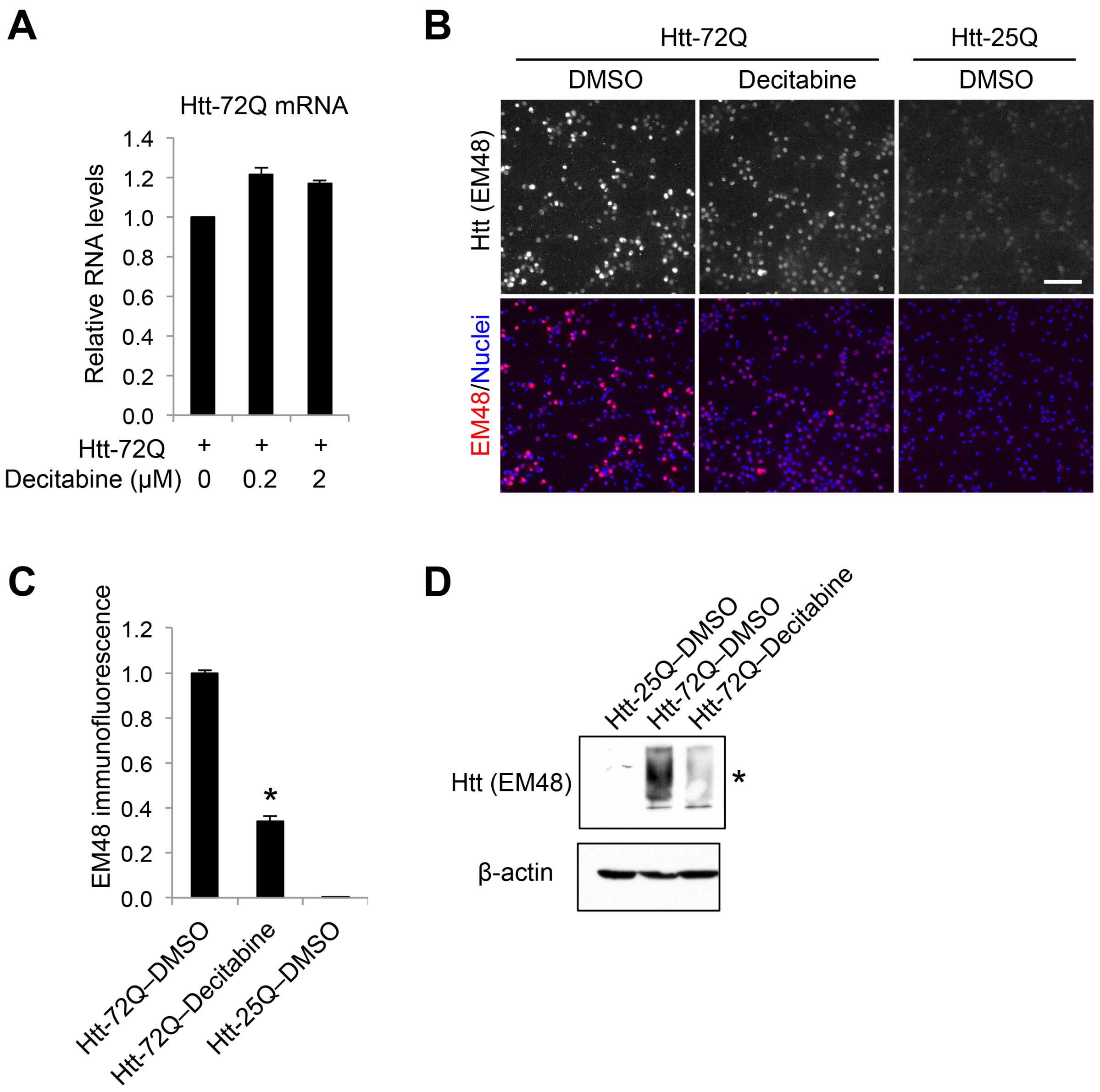

Figure S2. Inhibition of DNMTs attenuates mutant Htt aggregates in primary cortical neurons

(A) DIV 5 mouse primary cortical neurons were infected with Htt-72Q lentivirus and treated with decitabine or vehicle (DMSO). RNA was prepared at DIV 10 and subjected to qRT-PCR analysis for Htt-72Q (human). *β-actin* and *Hprt* were used as reference genes. Decitabine did not decrease the levels of Htt-72Q mRNA compared to vehicle (ANOVA, n = 6 independent experiments).

(B) DIV 5 cortical neurons were infected with Htt lentivirus and treated with decitabine (0.2 μM) or DMSO. Neurons were fixed at DIV 11-12 and subjected to indirect immunofluorescence with a specific mouse monoclonal Htt antibody EM48, which preferentially detects aggregated mutant protein. Nuclei were labeled with Hoechst 33342. Images were captured using an Operetta high-content imaging system (PerkinElmer) with a 20× objective lens. Bar, 100 μm.

(C) EM48 immunofluorescence intensity in (B) was quantified using an ImageJ-based macro. Decitabine significantly decreased the levels of mutant Htt aggregates in Htt-72Q-expressing primary cortical neurons (ANOVA, *$P < 0.0001$ vs. Htt-72Q–DMSO, n = 18 wells from 6 independent experiments).

(D) Cortical neurons transduced and processed as in (A) were directly harvested in SDS sample buffer at DIV 10 and subjected to immunoblotting with anti-Htt EM48 monoclonal antibody. Blot was reprobed with anti-β-actin antibody. Representative immunoblot from 3 independent experiments is shown. Decitabine could decrease the levels of aggregated high molecular weight mutant Htt in a stacking gel (*).

Data in (A) and (C) are presented as mean + SEM.

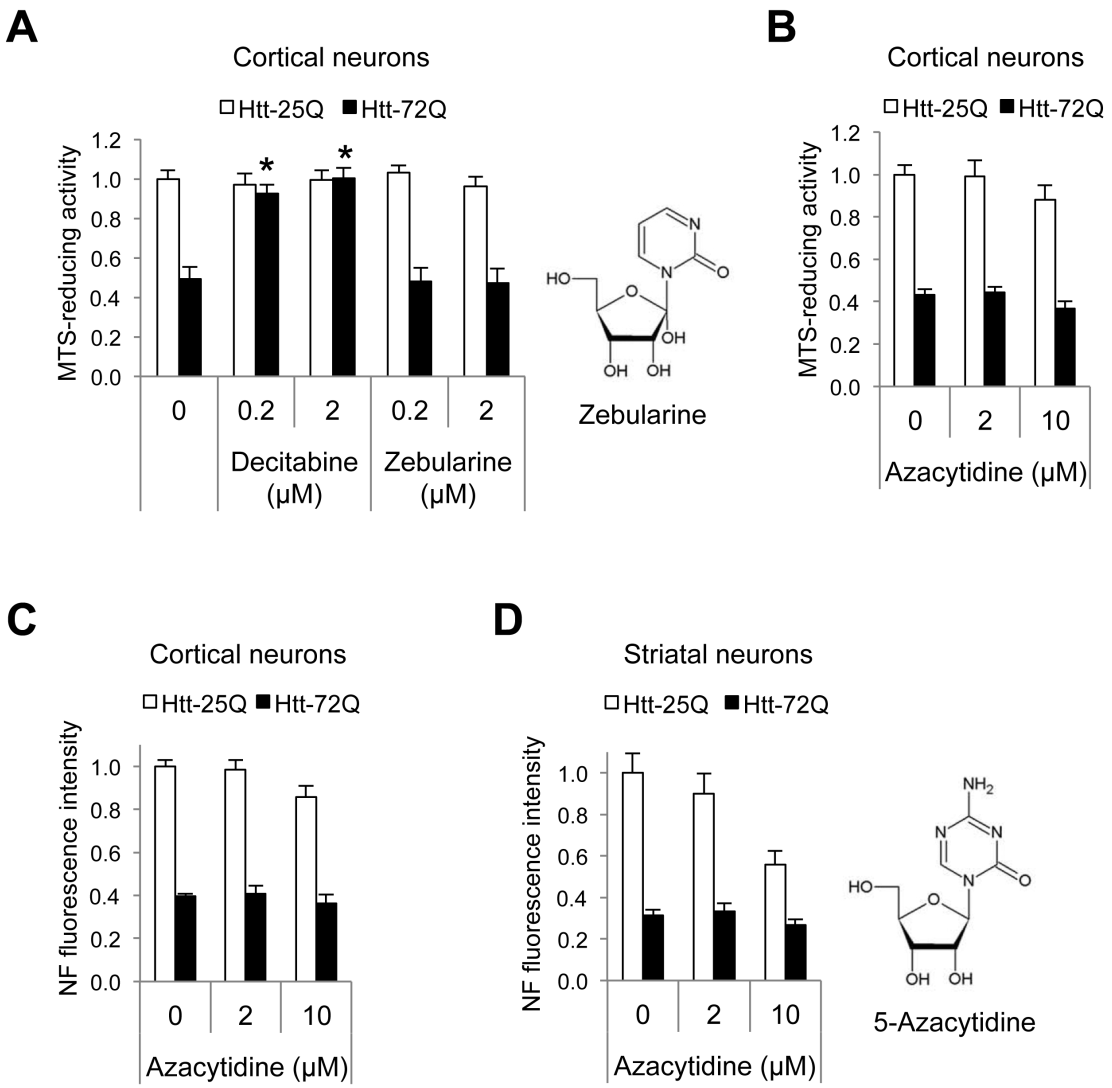

Figure S3. The effects of nucleoside analog DNMT inhibitors on mutant Htt-induced toxicity in primary neurons.

(A) DIV 5 cortical neurons transduced with lentivirus expressing Htt-72Q or Htt-25Q fragment were treated with the indicated drugs or DMSO (= 0 μM) and subjected to MTS assay at DIV 14. Decitabine, but not zebularine, increased the viability of mutant Htt-expressing neurons (ANOVA, *P < 0.0001 compared to Htt-72Q (0 μM); no significant difference in survival between zebularine and vehicle-treated Htt-72Q neurons; n = 8-18 wells per group, 3 independent experiments).

(B) Primary cortical neurons transduced and treated with 5-azacytidine were subjected to MTS assay as in (A). There was no significant difference in survival between 5-azacytidine- and vehicle-treated Htt-72Q neurons (ANOVA, n = 6-15 wells per group, 3 independent experiments).

(C) Primary cortical neurons were transduced and treated as in (A) and subjected to neurofilament (NF) immunofluorescence. There was no significant difference in NF immunofluorescence intensity between 5-azacytidine- and vehicle-treated Htt-72Q cortical neurons (ANOVA, n = 6-15 wells per group, 3 independent experiments)

(D) Primary striatal neurons transduced with Htt-25Q or Htt-72Q lentivirus at DIV 4 were treated with 5-azacytidine or DMSO. Seven days later, neurons were subjected to NF immunofluorescence. There was no significant difference in NF immunofluorescence intensity between 5-azacytidine- and vehicle-treated Htt-72Q striatal neurons (ANOVA, n = 9-17 wells, 3 independent experiments).

Data are presented as mean + SEM.

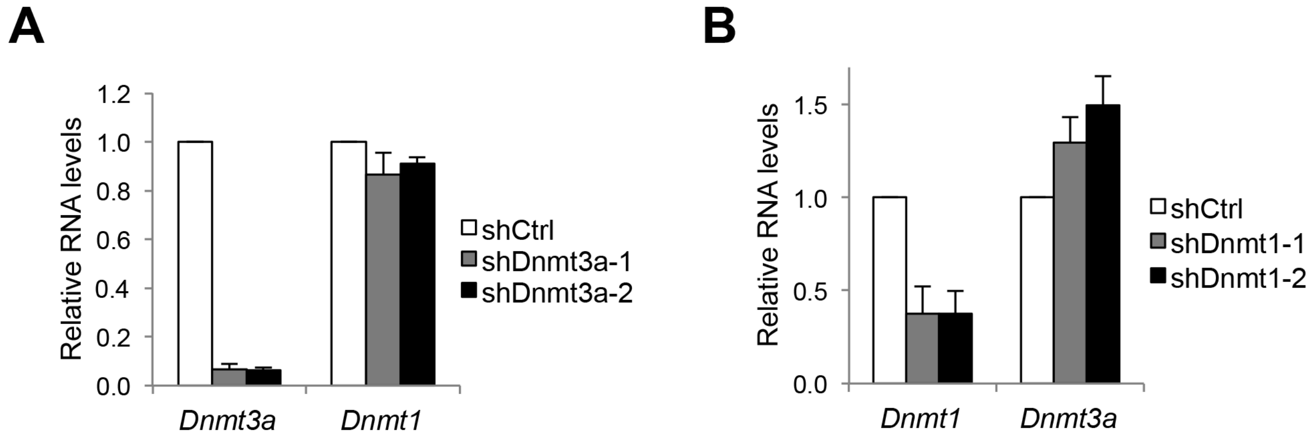

Figure S4. Knockdown of DNMT3A or DNMT1 did not decrease the expression of the other DNMTs.

(A) DIV 5 cortical neurons were transduced with lentivirus expressing *Dnmt3a* shRNA (shDnmt3a-1 or -2) or control shRNA along with Htt-72Q virus; 5 days later, RNA was prepared and subjected to qRT-PCR analysis. *Dnmt3a* RNAi did not significantly reduce the levels of *Dnmt1* mRNA in mutant Htt-expressing neurons, showing the specificity of these shRNAs (ANOVA, n = 3 independent experiments).

(B) Cortical neurons were transduced with lentivirus expressing *Dnmt1* shRNA (shDnmt1-1 or -2) or control shRNA along with Htt-72Q virus and processed as in (A). *Dnmt1* RNAi did not decrease the levels of *Dnmt3a* mRNA in mutant Htt-expressing neurons, showing the specificity of these shRNAs (ANOVA, n = 3 independent experiments).

Data are presented as mean + SEM.

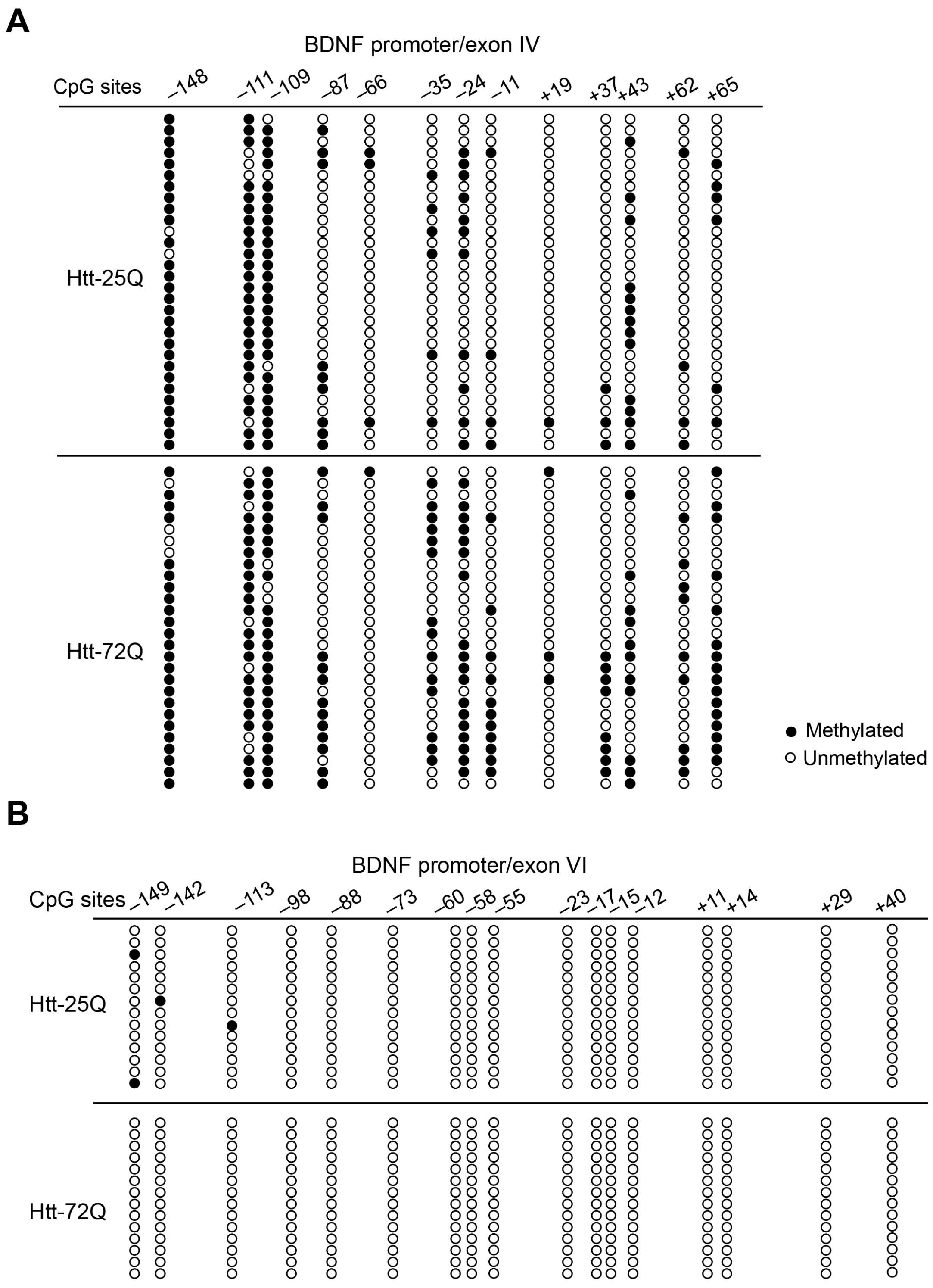

Figure S5. Analysis of CpG methylation status of *Bdnf* exon IV and VI regulatory regions in mutant Htt-expressing primary cortical neurons.

(A) Primary cortical neurons were transduced with Htt-25Q or Htt-72Q at DIV 5. Five days later, genomic DNA was prepared and subjected to bisulfite DNA sequencing analysis on the 13 CpG sites in the regulatory region of *Bdnf* exon IV. The positions of CpG sites are indicated relative to the TSS. Data are pooled from 7 independent experiments. Percentage of methylated cytosine residues was calculated and presented in Figure 4B.

(B) Primary cortical neurons were transduced and processed for bisulfite DNA sequencing analysis on the 17 CpG sites in the regulatory region of *Bdnf* exon VI as in (A). Data are pooled from 3 independent experiments. The CpG sites in this region were mostly unmethylated in Htt-25Q- and Htt-72Q-expressing neurons.

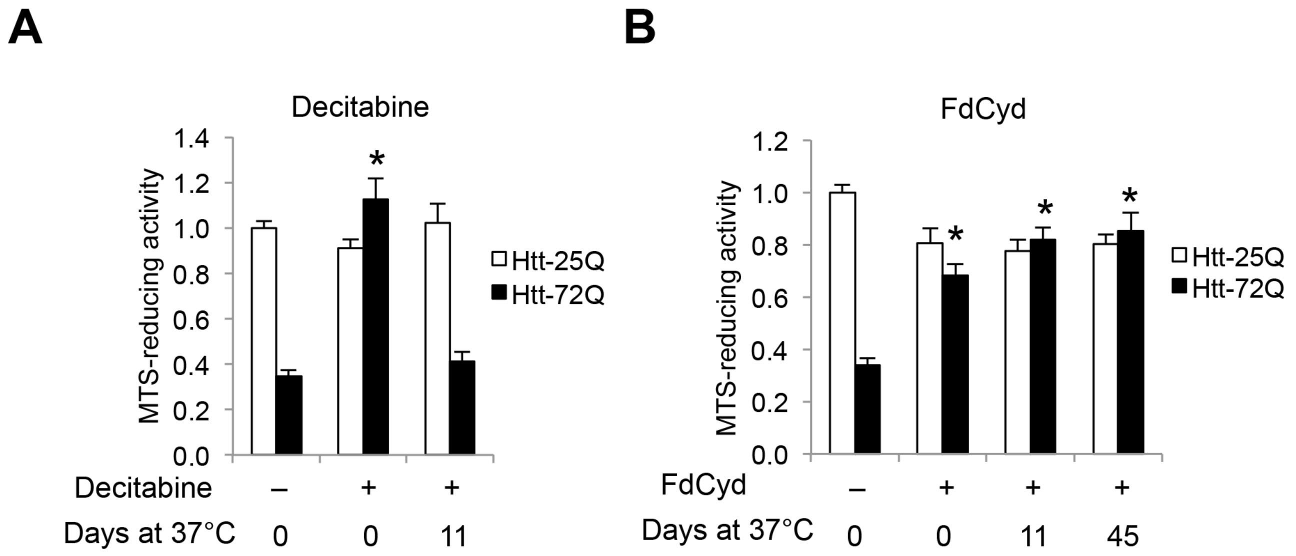

Figure S6. ***In vitro*** **stability of decitabine and FdCyd.**

(A) Decitabine (0.2 mM in saline) was first incubated at 37°C for indicated number of days *in vitro* and then added to the culture media at a final concentration 0.2 μM of DIV 5 primary cortical neurons transduced with Htt-25Q or Htt-72Q lentivirus. The neuroprotective activity of decitabine was tested using MTS assay at DIV 14. Decitabine lost its activity when tested after 11 days of incubation (ANOVA, $^*P < 0.0001$ compared to Htt-72Q plus vehicle, no difference between Htt-72Q plus vehicle vs Htt-72Q plus decitabine preincubated for 11 days, n = 9 wells, 3 independent experiments).

(B) Similar experiments were performed with FdCyd as in (A). In contrast to decitabine, FdCyd preincubated for 11 or 45 days showed neuroprotective effects comparable to FdCyd with no preincubation (ANOVA, $^*P < 0.0001$ compared to Htt-72Q plus vehicle, n = 9 wells, 3 independent experiments).

Data are presented as mean + SEM.

Table S1. List of compounds used in the epigenetic drug screen

1	CAY10433	HDAC inhibitor
2	Piceatannol	Resveratrol analog
3	CAY10591	SIRT1 activator
4	EX-527	SIRT1 inhibitor
5	SAHA	Calss I and II HDAC inhibitor
6	2-PCPA (hydrochloride)	LSD1 inhibitor
7	3-amino Benzamide	PARP inhibitor
8	SB 939	HDAC inhibitor
9	PCI 34051	HDAC8 inhibitor
10	4-iodo-SAHA	CalssI and II HDAC inhibitor (SAHA derivative)
11	Sirtinol	SIRT inhibitor
12	C646	HAT p300 inhibitor
13	Tubastatin A (trifluoroacetate salt)	HDAC6 inhibitor
14	Garcinol	p300 and PCAF HAT inhibitor
15	Ellagic Acid	Antioxidant; inhibitor of H3R17 methylation
16	Suberohydroxamic Acid (SBHA)	HDAC inhibitor
17	Apicidin	HDAC inhibitor
18	UNC0321 (trifluoroacetate salt)	G9a HMTase inhibitor
19	(-)-Neplanocin A	SAH hydrolase inhibitor (SAM-dependent MT inhibitor)
20	Cl-Amidine	PAD4 deiminase inhibitor
21	F-Amidine (trifluoroacetate salt)	PAD4 deiminase inhibitor
22	JGB1741	SIRT1 inhibitor
23	UNC0638	G9a HMTase inhibitor
24	Isoliquiritigenin	Antioxidant, anti-inflammatory, antitumor activities
25	CCG-100602	Inhibitor of Rho pathway-mediated signaling and activation of serum response factor transcription
26	CAY10669	pCAF (p300/CREB-binding protein-associated factor) HAT inhibitor
27	Zebularine	DNMT inhibitor
28	Delphinidin chloride	p300/CBP HAT inhibitor
29	Suramin (sodium salt)	SIRT1 inhibitor
30	Nicotinamide	SIRT inhibitor
31	2,4-Pyridinedicarboxylic Acid	Histone demethylase inhibitor
32	PFI-1	BET bromodomain inhibitor
33	5-Azacytidine	DNMT inhibitor
34	Decitabine	DNMT inhibitor
35	(+)-JQ1	BET bromodomain inhibitor
36	(-)-JQ1	Negative control for (+)-JQ1
37	BSI-201	PARP1 inhibitor
38	1-Naphthoic Acid	SIRT inhibitor
39	AG-014699	PARP-1 inhibitor
40	IOX1	inhibitor of 2-oxoglutarate oxygenases
41	MI-2 (hydrochloride)	Inhibitor of the menin-MLL fusion protein interaction
42	MI-nc (hydrochloride)	Weak inhibitor of the menin-MLL fusion protein interaction; negative control for MI-2
43	Lomeguatrib	O^6-Methylguanine-DNA methyltransferase (MGMT) inhibitor
44	Daminozide	Inhibitor of the human 2-oxoglutarate (JmjC) histone demethylases, KDM2A, PHF8, and KDM7A
45	GSK-J1 (sodium salt)	JMJD3 selective histone demethylase inhibitor
46	GSK-J2 (sodium salt)	Poor JMJD3 inhibitor (negative control)
47	GSK-J4 (hydrochloride)	JMJD3 selective histone demethylase inhibitor
48	GSK-J5 (hydrochloride)	Weak JMJD3 demethylase inhibitor (inactive control)

49	Valproic Acid (sodium salt)	HDAC inhibitor
50	Tenovin-1	p53 activator; SIRT1 and SIRT2 inhibitor
51	Tenovin-6	p53 activator; SIRT1, SIRT2, and SIRT3 inhibitor
52	Sodium Butyrate	HDAC inhibitor
53	Anacardic Acid	HAT (p300 and pCAF) inhibitor; anti-inflammatory, anti-tumor, molluscicidal, and anti-microbial activity
54	AGK2	SIRT2 inhibitor
55	CAY10603	HDAC6 inhibitor
56	Chaetocin	HMT inhibitor with selectivity for Lys9-HMTs (SU(VAR)3-9, G9a, DIM5)
57	Splitomicin	Sir2p HDAC inhibitor
58	CBHA	HDAC inhibitor,
59	M 344	HDAC inhibitor
60	Oxamflatin	HDAC inhibitor
61	Salermide	SIRT1 and SIRT2 inhibitor
62	Mirin	Mre11-Rad50-Nbs1 (MRN) inhibitor
63	Pimelic Diphenylamide 106	Calss I HDAC inhibitor
64	(S)-HDAC-42	HDAC inhibitor
65	MS-275	HDAC (HDAC1) inhibitor
66	RG-108	DNMT inhibitor
67	2',3',5'-triacetyl-5-Azacytidine	DNMT inhibitor
68	S-Adenosylhomocysteine (SAH)	Product of SAM-dependent methylation of DNA, RNA, and histones and other proteins
69	UNC0224	G9a HMTase inhibitor
70	Chidamide	HDAC inhibitor
71	3-Deazaneplanocin A	S-adenosyl-L-homocysteine hydrolase inhibitor; EZH2 inhibitor
72	Sinefungin	SET domain-containing methyltransferase inhibitor
73	N-Oxalylglycine	Inhibitor of α-ketoglutarate-dependent enzymes and prolyl hydroxylase domain-containing proteins PHD1 and PHD2
74	AMI-1 (sodium salt)	PRMTs inhibitor
75	UNC1215	L3MBTL3 domain inhibitor
76	trans-Resveratrol	Antioxidant, antiproliferative and anti-inflammatory activity, and cyclooxygenase-1 inhibitor
77	2,4-DPD	HIF-PH inhibitor
78	DMOG	HIF-PH inhibitor
79	Trichostatin A	HDAC inhibitor
80	CAY10398	HDAC1 inhibitor
81	SGC0946	DOT1L inhibitor
82	EPZ5676	DOT1L inhibitor
83	EPZ6438	EZH2 inhibitor
84	GSK126	EZH2 inhibitor

1-80: Epigenetic Screening Library from Cayman Chemical (Item Number 11076)

81-84: Xcessbio Biosciences Inc.

Table S2. List of primers used for experiments

(A) Primer sequences for mRNA analysis

Genes	Forward	Reverse
Dnmt3a	AATAGAGACCCTCGGAGGCA	CCTGCTGCTAGTTGGGTTCT
Dnmt1	AACAGCTCCAGCCCGAGT	TTTTCTGTTAAGCCATCTCTTTCC
Bdnf exon IX (protein coding)	GACAAGGCAACTTGGCCTAC	CGTGCTCAAAAGTGTCAGCC
Bdnf exon I	CCTGCATCTGTTGGGGAGAC	GCCTTGTCCGTGGACGTTTA
Bdnf exon II	CTAGCCACCGGGGTGGTGTAA	CGCCTTCATGCAACCGAAGT
Bdnf exon III	GCTTCATTGAGCCCAGTTCC	GCCTTGTCCGTGGACGTTTA
Bdnf exon IV	CAGAGCAGCTGCCTTGATGTT	GCCTTGTCCGTGGACGTTTA
Bdnf exon VI	TTGGGGCAGACGAGAAAGCGC	AGGATGGTCATCACTCTTCTC
18s	AGTCGGCATCGTTTATGGTC	CGAAAGCATTTGCCAAGAAT
Hprt	TTGACACTGGTAAAACAATGCAAAC	GAGAGGTCCTTTTCACCAGCA
β-actin (Actb)	AGTGTGACGTTGACATCCGTA	GCCAGAGCAGTAATCTCCTTCT
Drd2	CTGGAGCCAAAAGCAGTCTG	TCCTTCAGGTTTCCGACGCC
Ppp1r1b	CCAACCCCTGCCATGCTTT	TTGGGTCTCTTCGACTTTGGG
Penk	TGGCGTAGGGCCTGCGTC	TGTAAAGCGGCCGCGTCG
Pcp4	CGACCAACGGAAAAGACAAG	TGTCTCTGGTGCATCCATGT
Rasd2	AACTGCGCCTACTTCGAGG	GGTGAAAAGCATCGCCGTACT
Kdm8	GCTGGACCTCGGTGAGAAG	TCCCAGGAGTAGTCTAGGACG
Adora2A	GCCATCCCATTCGCCATCA	GCAATAGCCAAGAGGCTGAAGA
human HTT exon1	TCAACCTCCTCCACAGGCAC	AGGCTCCTCAGCCACAGCT

(B) Primer sequences for bisulfite sequencing analysis

Genes	Forward	Reverse
Bdnf IV	GTGAATTTGTTAGGATTGGAAGTGAAAATA	CTAAACAAAAACTAAAAAATTTCATACTAACTC
Bdnf VI	GGTAGGTATAGAGTTTTGGGTTTAAGTAG	ACACTAAAATCAAACATTATTTAACTCTTC

(C) Primer sequences for MeDIP analysis

Genes	Forward	Reverse
Bdnf IV	GCGCGGAATTCTGATTCTGGTA	CTGCCTTGACGTGAGCTGTC
Gapdh	CTCTGCTCCTCCCTGTTCC	TCCCTAGACCCGTACAGTGC

(D) Primer sequences for ChIP analysis

Genes	Forward	Reverse
Bdnf IV	CTTCTGTGTGCGTGAATTTGCT	AGTCCACGAGAGGGCTCCA
